# Is There a Relationship between the Elasticity of Brain Tumors, Changes in Diffusion Tensor Imaging, and Histological Findings? A Pilot Study Using Intraoperative Ultrasound Elastography

**DOI:** 10.3390/brainsci11020271

**Published:** 2021-02-21

**Authors:** Santiago Cepeda, Sergio García-García, María Velasco-Casares, Gabriel Fernández-Pérez, Tomás Zamora, Ignacio Arrese, Rosario Sarabia

**Affiliations:** 1 Department of Neurosurgery, University Hospital Río Hortega, 47012 Valladolid, Spain; segarciagarc@saludcastillayleon.es (S.G.-G.); iarreser@saludcastillayleon.es (I.A.); rsarabia@saludcastillayleon.es (R.S.); 2 Department of Radiology, University Hospital Río Hortega, 47012 Valladolid, Spain; mvelascoca@saludcastillayleon.es (M.V.-C.); gcfernandez@saludcastillayleon.es (G.F.-P.); 3 Department of Pathology, University Hospital Río Hortega, 47012 Valladolid, Spain; tzamorama@saludcastillayleon.es

**Keywords:** brain tumor, ultrasound, elastography, diffusion tensor imaging, Ki67 index

## Abstract

Intraoperative ultrasound elastography (IOUS-E) is a novel image modality applied in brain tumor assessment. However, the potential links between elastographic findings and other histological and neuroimaging features are unknown. This study aims to find associations between brain tumor elasticity, diffusion tensor imaging (DTI) metrics, and cell proliferation. A retrospective study was conducted to analyze consecutively admitted patients who underwent craniotomy for supratentorial brain tumors between March 2018 and February 2020. Patients evaluated by IOUS-E and preoperative DTI were included. A semi-quantitative analysis was performed to calculate the mean tissue elasticity (MTE). Diffusion coefficients and the tumor proliferation index by Ki-67 were registered. Relationships between the continuous variables were determined using the Spearman ρ test. A predictive model was developed based on non-linear regression using the MTE as the dependent variable. Forty patients were evaluated. The pathologic diagnoses were as follows: 21 high-grade gliomas (HGG); 9 low-grade gliomas (LGG); and 10 meningiomas. Cases with a proliferation index of less than 10% had significantly higher medians of MTE (110.34 vs. 79.99, *p* < 0.001) and fractional anisotropy (FA) (0.24 vs. 0.19, *p* = 0.020). We found a strong positive correlation between MTE and FA (*r*_s_ (38) = 0.91, *p* < 0.001). A cubic spline non-linear regression model was obtained to predict tumoral MTE from FA (*R*^2^ = 0.78, *p* < 0.001). According to our results, tumor elasticity is associated with histopathological and DTI-derived metrics. These findings support the usefulness of IOUS-E as a complementary tool in brain tumor surgery.

## 1. Introduction

The biomechanical properties of tissues and their histological correlations have received increased attention in recent years. There are several studies in which the primary objective was to determine in vivo tissue elasticity changes that occur under pathological conditions. To reveal these changes, two main radiological techniques have been applied: magnetic resonance elastography (MRE) and ultrasound techniques, such as shear wave and strain elastography. There seems to be a strong correlation between elastic characteristics and histological features, especially for liver, prostate, thyroid, and breast neoplasms, using elasticity as a discriminatory parameter [1,2,3]. 

Regarding brain tumors, the application of intraoperative ultrasound elastography (IOUS-E) has made it possible to determine the elasticity values of tumors and the correlation between histological types and grades of malignancy [4,5,6,7,8,9,10,11].

Diffusion tensor imaging (DTI) is another tool that has noticeably evolved in recent years. Its application in neuro-oncology has extended beyond pre-surgical planning through fiber tracking [12], and it offers a wide variety of assessment tools for the quantitative and qualitative analysis of brain tumors and perilesional white matter [13].

Because IOUS-E is a relatively new imaging technique, before expanding its application as a tool in brain tumor surgery, it is necessary to establish the associations between brain tumor elasticity and other parameters previously described as diffusion coefficients and cell proliferation. By establishing links between tumor elasticity and other biological parameters, new models of tumor infiltration and resection boundaries may be developed in the future. Additionally, it may be possible to take advantage of the information that this intraoperative imaging modality provides in real time. Hence, the objective of the present study is to establish the correlations mentioned above.

## 2. Materials and Methods

Data regarding intraoperative ultrasound images in 12 of the 40 included patients have been previously reported [11]. The prior study dealt with IOUS-E features in brain tumors, whereas in this study, we report a correlation analysis of the tumor elasticity, DTI-derived metrics, and the cell proliferation index.

### 2.1. Patient Selection

**Figure 1:**
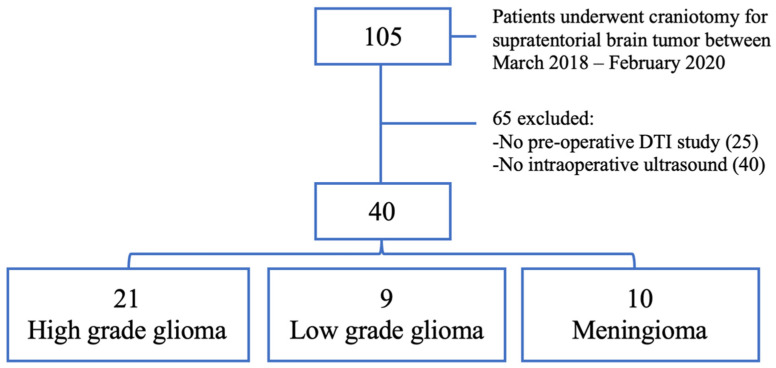
Flowchart showing the patient selection criteria.

A retrospective analysis was performed based on patients consecutively admitted at our center for diagnosed supratentorial brain tumors between March 2018 and February 2020. All patients who underwent surgery by craniotomy had an IOUS-E study and preoperative DTI were included (Figure 1). 

### 2.2. Histological Technique

Tumor samples were randomly selected for histological evaluation. Hematoxylin and eosin-stained sections were used to confirm the histopathological diagnosis. Ki-67 was immunohistochemically detected using a monoclonal antibody (Clone MIB-1, Monoclonal Mouse, Anti-Human, IgG1, Dako Corp., Carpinteria, CA, USA). The proliferation index was defined as the number of MIB-1-positive tumor cells from the total number of tumor cells observed under a light microscope at × 200 magnification. A high proliferative index was defined as a Ki-67 value greater than 10%, according to previous publications [14].

### 2.3. Acquisition and Image Processing

All subjects were scanned using a 1.5 T magnetic resonance imaging (MRI) scanner (Signa HDxt; GE Healthcare, Milwaukee, WI, USA). MRI sequences included axial T1-weighted (T1W) and post-contrasted T1W spin-echo with the following parameters: TR/TE, 7.98 ms/2.57 ms; FOV, 220 × 220 mm; matrix, 512 × 512; slice thickness, 1 mm; axial T2-weighted (T2W) fast spin-echo: TR/TE, 5220 ms/96.12 ms; FOV, 220 × 220 mm; matrix, 512 × 512; and slice thickness, 5 mm. DTI data were acquired using a single-shot echo-planar imaging sequence (TE, 96 ms; TR, 13675 ms; FOV, 256 × 256 mm; matrix, 128 × 128; voxel size, 1015 × 1015 × 3 mm). The diffusion-weighting gradient was applied in 25 isotropically distributed directions using a b value of 1000 s/mm^2^. Fifty gapless slices were obtained to cover the whole brain, with a thickness of 3 mm. 

**Figure 2:**
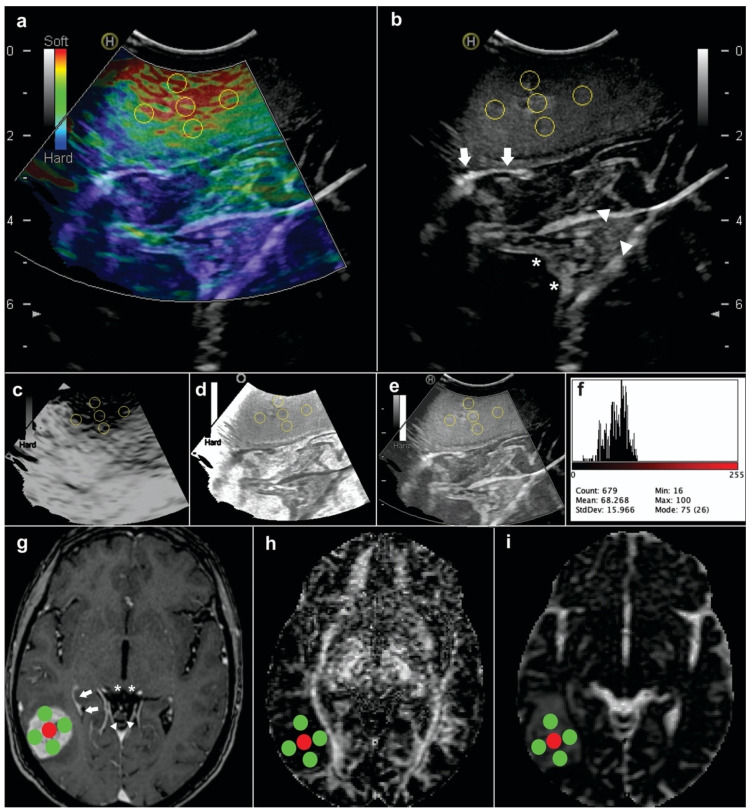
Illustrative case showing the methodology applied for image analysis. Images correspond to a 72-year-old woman with a right parieto-occipital glioblastoma. Regions of interest (ROIs) were placed in the central/core and peripheric tumor regions that were revealed using (**a**) elastograms and (**b**) B-mode images. Ultrasound (US) images were decomposed into a hue-saturation-brightness (HSB) type: (**c**) hue; (**d**) saturation; and (**e**) brightness mode. (**f**) Mean tissue elasticity (MTE) values were calculated using histogram intensity analysis. (**g**) The axial T1-post contrast image was selected based on anatomical references: Inferior colliculus (white asterisk); occipital horn of lateral ventricle (white arrows); and cerebellar vermis (white head arrows). These structures were also visualized in US images. Tumoral values were calculated using diffusion maps of the (**h**) fractional anisotropy and (**i**) mean diffusivity.

The tumor volume was determined through semi-automated segmentation tools (Elements, Brainlab AG, Munich, Germany). A Hitachi Noblus model ultrasound with a C42 probe and a frequency range of 4 to 8 MHz was used for intraoperative tumor evaluation. The ultrasound used a 20 mm (radius) scan width and an 80° field of view (FOV) scan angle. Ultrasound images were acquired after craniotomy and before dural opening. After the tumor was localized in B-mode, the images were acquired in elastography mode. Strain elastography only provides qualitative information through a color map. The color scale of these images assigns a value ranging from 0 (red = soft) to 256 (blue = hard) that is constant across all acquisitions. Elastograms were acquired in different projections and stored in Digital Imaging and Communication On Medicine (DICOM) format for offline postprocessing. Following previous studies [11,15], we performed a semi-quantitative analysis to compute numerical values from the elastogram. Using ImageJ software version 1.50i (National Institutes of Health, MA, USA), we performed a decomposition of the image into HSB format (hue-saturation-brightness). We positioned the regions of interest (ROI), each approximately 20 pixels in diameter, in the areas defined as the tumor core and intra-tumoral periphery (Figure 2). The assessment of tumor elasticity using ROIs and its interobserver variability has been described in a previous publication [11]. The mean tissue elasticity (MTE) corresponded to the average values of the different pixels and was calculated from the histogram of intensities expressed in arbitrary units [16].

MRI was processed using the DSI Studio program (dsi-studio.labsolver.org (accessed on 1 April 2020)). Maps of the diffusion coefficients were calculated including the fractional anisotropy (FA), mean diffusivity (MD), axial diffusivity (AD), and radial diffusivity (RD). Co-registered images in T1W post gadolinium (high-grade gliomas and meningiomas) and T2W (low-grade gliomas) were used to place ROIs (diameter = 5 mm/voxels = 60) following the same location as that in the ultrasound images (Figure 2). Anatomical structures were taken as references, and the neuronavigation system with the screen capture function was used simultaneously as a guide for analyzing ultrasound (US) and magnetic resonance (MR) images with as much correspondence as possible. The average values of each diffusion coefficient and MTE were calculated for the core region, intra-tumoral periphery, and whole tumor volume. Image selection and measurements were conducted by a neurosurgeon with experience in neuroimaging (S.C.), supervised by two senior radiologists (G.F. and M.V.).

### 2.4. Statistical Analysis

The distribution of the continuous variables was assessed using the Shapiro–Wilk test. To establish differences between histopathological groups, the Kruskal–Wallis test was used, and epsilon squared (*ε*^2^) was calculated to measure the effect size. In the post hoc analysis, the Dunn method with Holm correction was applied. The effect sizes of comparison groups that reached statistical significance were obtained using the Wilcoxon Rank Sum Test. MTE values and DTI-derived metrics were compared with a high proliferation index, defined as >10% using the Wilcoxon–Mann–Whitney test, calculating the *U* statistic and the effect size with the r coefficient. The relationships between the quantitative variables were calculated using the Spearman *ρ* test. 

After establishing the correlations between the tumor elasticity and DTI-derived metrics, a predictive model was developed, using the MTE as the dependent variable and the diffusion coefficients as predictors. The collinearity diagnosis between predictors was performed using the variance inflation factors measure (VIF) and the Farrar–Glauber test. Models were based on linear, logarithmic, quadratic polynomial, and cubic spline regression. Assumptions of homogeneity of variance, linearity, and the distribution of residuals were verified for each model. For model validation, the study sample was divided in a randomized manner, applying an 80/20 ratio to fit the models; the remaining 20% was used for validation. The estimators used for the assessment of the models were as follows: the determination coefficient (R squared); the absolute fit of the model represented by the mean absolute error (MAE); and efficiency estimators, such as the index of agreement (IOA) [17] and coefficient of efficiency (COE) [18].

All statistical analyses were performed using the R version 4.0.0 program (R Foundation for Statistical Computing, Vienna, Austria), with *p* < 0.05 considered to indicate a statistically significant difference.

## 3. Results

One hundred and five patients underwent surgery during the study period; 40 patients were excluded due to the lack of an intraoperative elastographic study, while 25 were excluded due to the absence of a pre-surgical DTI study. Therefore, 40 patients met the selection criteria and were available for analysis.

Twenty-one patients were women (52.5%) and 19 were men (47.5%). The mean age was 59.43 ± 12.13 years. The pathological diagnoses were as follows: 21 high-grade gliomas (HGG); 9 low-grade gliomas (LGG); and 10 meningiomas. The clinical characteristics of the sample are summarized in Table 1.

**Figure 3:**
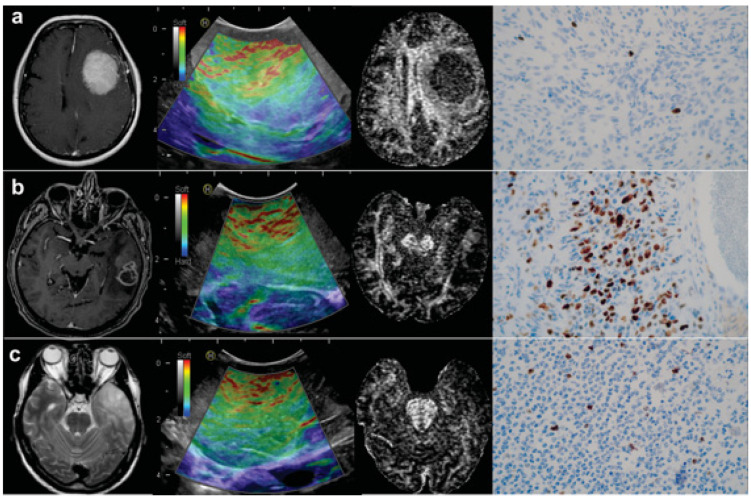
Illustrative cases of histopathological groups: (**a**) 75-year-old woman with a left frontal meningioma; (**b**) 56-year-old man with a left temporal low-grade glioma; and (**c**) 54-year-old man with a left temporal glioblastoma. From left to right: Axial T1-weighted post contrast slices for glioblastoma and meningioma and T2-weighted for low-grade glioma; ultrasound elastography images; fractional anisotropy maps; and immunohistochemical staining of Ki-67 at × 40 magnification.

Statistical analysis was performed based on the values corresponding to the whole tumor, defined as the average of the core area and the intra-tumoral periphery. There were significant differences in the elasticity values, diffusion coefficients, and proliferation index between the histopathological groups (Supplementary Figure S1). MTE values were significantly lower in high-grade gliomas (HGG) and low-grade gliomas (LGG) than in meningiomas (H = 13.96; *df* = 2; *p* < 0.001). The FA showed the same tendency, with lower values for gliomas than meningiomas (H = 16.10; *df* = 2; *p* < 0.001). The coefficients mean diffusivity (MD), axial diffusivity (AD,) and radial diffusivity (RD) exhibited a similar pattern, with higher values for gliomas. These differences reached statistical significance. None of the analyzed variables displayed differences between LGG and HGG. Regarding Ki-67, HGG had higher values than LGG and meningiomas, but there were no differences between the last two groups mentioned. The results of the descriptive and statistical analysis are detailed in Table 2 and Supplementary Table S1. Illustrative cases of the three tumor types and their radiological and histological patterns are shown in Figure 3.

After the dichotomizing of the Ki-67 variable into high (>10%) and low (<10%), it was observed that the cases with a proliferation index of less than 10% had significantly higher medians of MTE and FA (Table 3).

**Figure 4:**
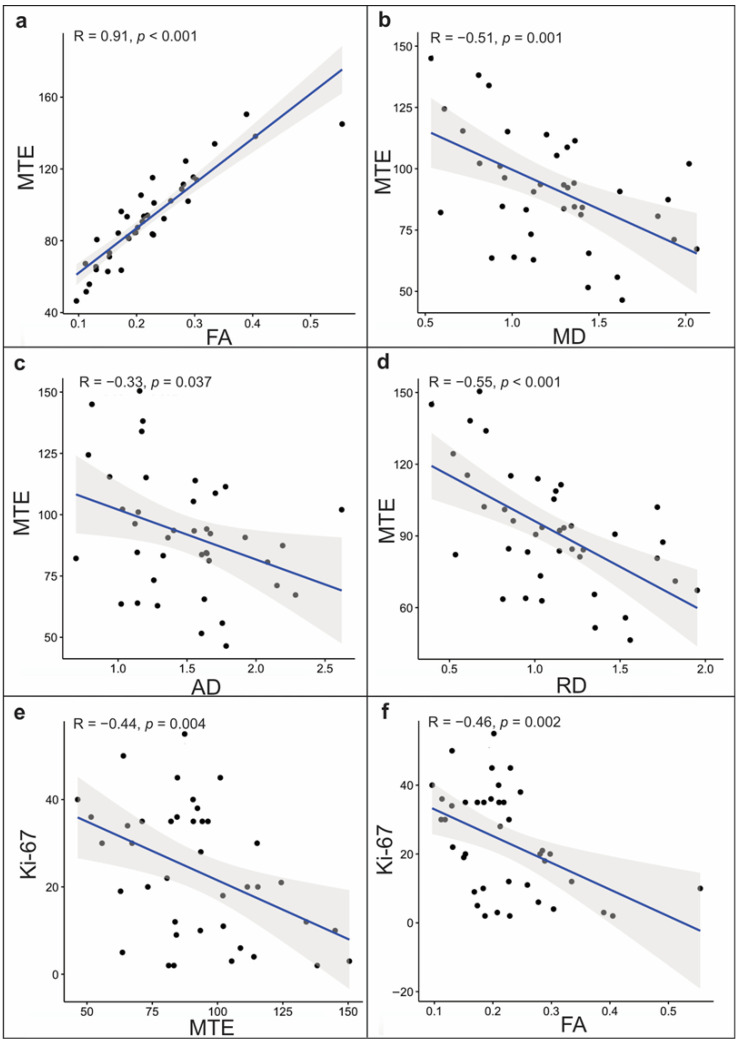
Scatterplots of a correlation analysis of the whole tumor volume for the mean tissue elasticity (MTE) and (**a**) fractional anisotropy, (**b**) mean diffusivity, (**c**) axial diffusivity, and (**d**) radial diffusivity. Significant correlations were also found between Ki-67, (**e**) MTE, and (**f**) fractional anisotropy.

The correlations between the continuous variables were determined by the Spearman ρ. Regarding total tumor volume, we found a strong positive correlation between MTE and FA (*r*_s_ (38) = 0.91, *p* < 0.001). The MD and RD exhibited a moderate negative correlation with MTE (*r*_s_ (38) = −0.51; *p* = 0.001; *r*_s_ (39) = −0.55, *p* < 0.001) and a weak negative correlation for the AD and MTE (*r*_s_ (38) = −0.33, *p* = 0.037). The Ki-67 displayed a weak significant negative correlation with MTE and FA (*r*_s_ (38) = −0.44, *p* = 0.004; *r*_s_ (38) = −0.46, *p* = 0.002) (Figure 4).

**Figure 5:**
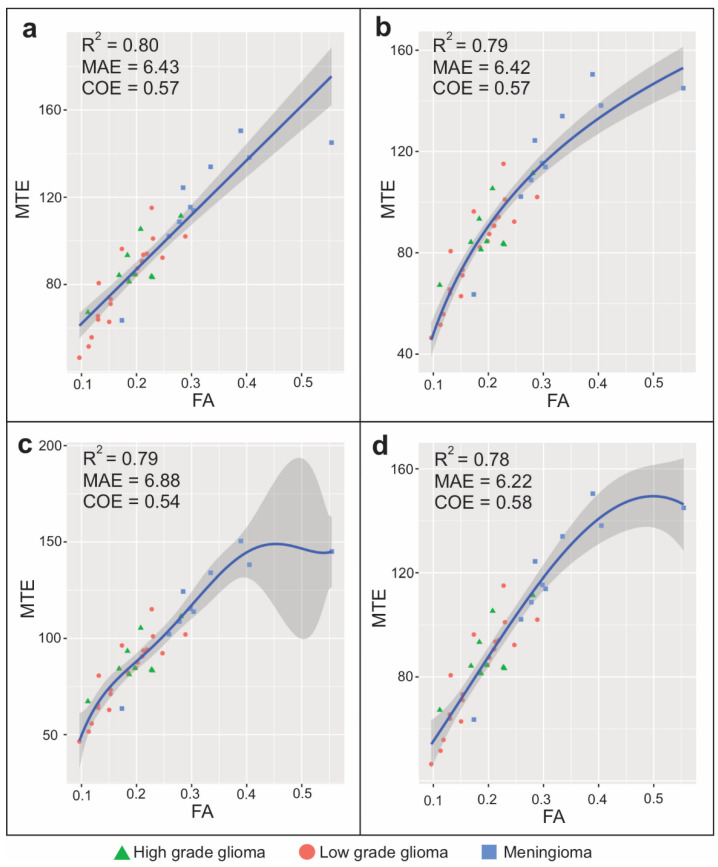
Model fit represented by scatterplots of (**a**) linear, (**b**) logarithmic, (**c**) quadratic polynomial, and (**d**) cubic spline regressions. The Y-axis shows the mean tissue elasticity as the dependent variable and the X-axis shows fractional anisotropy values as the predictor. Gray zone represents a 95% confidence interval. In the left upper corner of each model, the adjusted *R*^2^, mean absolute error (MAE), and coefficient of efficiency (COE) are shown. Cases are presented by histopathologic groups detailed in the legend at the bottom of the figure.

We obtained a preliminary predictive model for tumor elasticity based on all the DTI-derived metrics. The collinearity diagnosis showed that the variables MD, AD, and RD exhibited a high correlation between each other and a suppression effect between each other in the model (Supplementary Figure S2). After applying a variable selection algorithm using stepwise regression, the use of the FA as the sole predictor was established. The linear regression model did not meet the assumptions of homoscedasticity of variance. The data of the non-linear models met the assumptions of homogeneity of variance and linearity, while the residuals were approximately normally distributed (Supplementary Table S2 and Supplementary Figure S3). After the validation process, different estimators for the assessment of the models were calculated (Supplementary Table S3 and Figure 5).

The cubic spline regression model (F(6,33) = 44.28; *p* < 0.001; *R*^2 =^ 0.78; MAE = 6.22; IOA = 0.79; COE = 0.58) showed the best performance in terms of the efficiency, accuracy, and predictive capacity.

## 4. Discussion

In the present study, we found that the elastography values and tensor-derived metrics were significantly different across tumor groups (gliomas and meningiomas). After correlation analysis, we observed that the elasticity values of brain tumors evaluated by IOUS-E were positively correlated with the FA and inversely correlated with the Ki-67 index. The regression analysis allowed us to calculate the value of the MTE from the tumor FA with a predictive capacity close to 80%. Our findings suggest that tumor elasticity measured by IOUS-E could be useful in the characterization of different tumor types. Furthermore, the elasticity values could be associated with the tumor cytoarchitecture represented by the diffusion of water molecules and the proliferation index.

Among the strengths of our work, we can mention that we followed a rigorous methodology for the image acquisition and processing. Furthermore, we included a representative sample of brain tumors with different histological behavior, which allowed us to search for patterns in each technique used. There is no precedent regarding the use of IOUS-E and its correlation with the diffusion tensor or histological findings. Therefore, the results of this quantitative analysis are the most notable aspect of our study. 

In addition to its retrospective nature and sample size, the main limitation of our work is the lack of an exact correspondence between the ultrasound and magnetic resonance images and the samples analyzed by histological techniques. In other words, proper correspondence between the tumor sample and DTI values might only be achieved during stereotactic or navigated biopsies. Therefore, we are aware of the margin of error that could exist in the comparison. Considering this, beyond analyzing the data for a specific region, we used the averages to obtain a global approximation of the entire tumor volume. Therefore, the diffusion coefficients and elasticity values revealed a pattern for each histological type, even in the presence of intra-tumoral heterogenicity. Another limitation is that we did not evaluate the peritumoral region and adjacent normal white matter because the lack of correspondence between the measurement techniques tends to increase as attempts are made to try and evaluate regions located far from a well-defined area—in this case, the tumor.

Some publications have evaluated the elastic capacity of brain tissue via cadaveric studies, and present variable results [19,20,21]. MRE was recently demonstrated to have the ability to analyze the regional mechanical properties of the brain in vivo [22,23] and can be used in the characterization of brain tumors [24,25,26]. IOUS-E offers the possibility of analyzing tumors and their elastic properties, and can be applied as a tool during tumor resection. According to previous publications [4,6,8,9,10,11], gliomas were found to have lower elastic/stiffness values than meningiomas, while differences could also be observed in the peritumoral regions between histopathological groups [11]. These findings are consistent with those found in our series. 

Regarding elastography and its correlation with histology, publications are scarce. There are descriptions based on cadaveric studies of the normal brain; some find a negative relationship between the shear modulus and the total number of cell nuclei in areas of the normal brain [19]. Similar results were found by Wang et al. [27], who described an inverse relationship between FA and Ki-67 in a glial infiltration model in rats. 

Concerning the comparison of elasticity and tensor-derived metrics, a positive relationship between FA and shear modulus values was described by Johnson et al. [28] in an MRE-based study of the areas of a normal brain. Meanwhile, in the work of Budday et al. [19], an inverse relationship was found. Regarding MD, a negative relationship has been described between MD and hippocampal stiffness measured in patients diagnosed with Alzheimer’s disease using MRE [29]. 

In our study, the values of the diffusion coefficients in the histopathological groups showed a similar trend to those of several previous publications [30,31,32,33], in which HGG obtained lower FA values compared to other tumor types. Regarding MD values, significantly higher values were observed for gliomas, especially LGG, compared to meningiomas. These data corroborate the histopathological nature of this type of neoplasm, with increased cellularity, invasion, and disruption of normal white matter. 

In our investigation, statistical analyses revealed that, despite the lack of biological correspondence between FA and MD, and while these coefficients measure two different aspects of diffusivity, there was a strong and significant correlation between them. These results agree with those of previous studies [30,31]. 

The relationship between diffusion coefficients and cellularity is an issue that remains controversial. Our results agree with previous publications in which tumor cellularity showed a positive correlation with FA and an inverse relationship with MD [30,33]. However, other authors have found opposite results [34,35]. These differences occur due to the diversity of measurement techniques, image processing, and histological sampling. For this reason, it is essential to establish a precise and reproducible methodology. According to our results and the conclusions reached by the authors of the studies mentioned above, we propose that due to the invasive nature of gliomas, the directionality of the diffusion and its magnitude are altered by the destruction of white matter. At the same time, other factors also contribute to the configuration of its radiological profile, such as the increase in extracellular space and the simultaneous increase in cellularity secondary to tumor growth. 

In the present study, we found significant associations between tumor elasticity and two widely documented variables, including DTI-derived metrics and the Ki-67 index. We can conclude that more aggressive tumors, such as HGG, tend to be softer, with lower FA and higher Ki-67 values. This link can be explained by a combination of white matter disruption, an increase in the extracellular space, and augmented tumor cell proliferation. In addition to its portability, cost, and versatility, IOUS-E could become a valuable intraoperative tool in brain tumor surgery due to the biological information provided by tissue elasticity. A preoperative DTI with all quantitative information correlated to suspected tumor malignancy could initially outline the extent of the planned resection. Intraoperative MTE tumor patterns may confirm, expand, or even update those limits. Therefore, our group is working on the correlations between these imaging tools to push current preoperative planning boundaries. 

The ultimate objective would be to incorporate IOUS-E as a new image modality within the neurosurgical armamentarium. In order to achieve this goal, it is essential to carry out prospective studies, including fully integrated navigation software and more accurate histological representations. This will enable us to expand this analysis to peritumoral areas. Consequently, the acquisition of elastography images could contribute to the elaboration of tumor infiltration maps and may also help to establish new resection limits. Therefore, we could take advantage of the real-time information that intraoperative ultrasound elastography provides and the availability of this technique in most centers.

## 5. Conclusions

According to our results, there is a significant relationship between elastographic values, coefficients obtained using the DTI, and the cell proliferation index. Our findings could serve as a basis to support elastography as a new technique in image-guided brain tumor surgery.

## Figures and Tables

**Table 1:** Patient characteristics.

Variable	*n*
Age	59.43 ± 12.13
Sex	
Female	21 (52.5%)
Male	19 (47.5 %)
Preoperative KPS	82.27 ± 10.93
Histopathology	
High-grade gliomas	
Glioblastoma	19 (47.5%)
Anaplastic astrocytoma grade III	1 (2.5%)
Anaplastic oligodendroglioma grade III	1 (2.5%)
Low-grade gliomas	
Astrocytoma grade II	4 (10%)
Oligodendroglioma grade II	5 (12.5%)
Meningiomas	
Meningioma grade I	8 (20%)
Meningioma grade II	2 (5%)
Tumor location	
Frontal	25 (62.5%)
Parietal	6 (15%)
Temporal	6 (15%)
Occipital	1 (2.5%)
Insular	2 (5%)
Initial volume (cm^3^)	38.64 ± 31.08

Values are expressed as the mean±standard deviation or as the frequency (%). KPS, Karnofsky Performance Score, and IQR, interquartile range.

**Table 2:** Univariate analysis of quantitative variables of the whole tumor region and histopathological groups.

Variable	AP	Descriptive Statistics				Kruskal–Wallis Test					Post hoc Dunn Test		Wilcoxon Rank Sum Test		
		*n*	Median	IQR	95% CI	*χ* ^2^	df	*p*	*ε* ^2^	95% IC	Comparison	*p* (Holm)	Z	*p*	*r*
MTE						13.96	2	<0.001	0.36	0.10–0.64					
	High-grade glioma	21	84.62	28.06	71.11–92.29						HGG-LGG	0.541			
	Low-grade glioma	9	84.22	10.12	81.28–105.40						HGG-MENINGIOMA	<0.001	3.59	<0.001	0.65
	Meningioma	10	119.90	27.08	108.76–139.49						LGG-MENINGIOMA	0.021	2.69	0.006	0.62
FA						16.10	2	<0.001	0.41	0.18–0.66					
	High-grade glioma	21	0.18	0.08	0.15–0.21						HGG-LGG	0.549			
	Low-grade glioma	9	0.20	0.04	0.17–0.23						HGG-MENINGIOMA	<0.001	3.85	<0.001	0.69
	Meningioma	10	0.30	0.10	0.27–0.39						LGG-MENINGIOMA	0.011	2.94	0.002	0.67
MD						13.85	2	<0.001	0.36	0.15–0.60					
	High-grade glioma	21	1.32	0.61	1.11–1.60						HGG LGG	0.607			
	Low-grade glioma	9	1.36	0.10	1.26–1.40						HGG-MENINGIOMA	0.002	3.29	0.001	0.59
	Meningioma	10	0.82	0.14	0.71–1.03						LGG-MENINGIOMA	0.002	3.27	<0.001	0.75
AD						9.64	2	0.008	0.25	0.07–0.52					
	High-grade glioma	21	1.60	0.58	1.26–1.76						HGG-LGG	0.419			
	Low-grade glioma	9	1.64	0.11	1.55–1.78						HGG-MENINGIOMA	0.019	2.58	0.009	0.46
	Meningioma	10	1.09	0.22	0.92–1.30						LGG-MENINGIOMA	0.012	2.86	0.003	0.66
RD						15.27	2	<0.001	0.39	0.19–0.60					
	High-grade glioma	21	1.15	0.58	1.01–1.47						HGG-LGG	0.602			
	Low-grade glioma	9	1.17	0.12	1.11–1.28						HGG-MENINGIOMA	0.001	3.47	<0.001	0.62
	Meningioma	10	0.69	0.18	0.57–0.87						LGG-MENINGIOMA	0.001	3.43	<0.001	0.79
Ki-67						22.04	2	<0.001	0.57	0.39–0.73					
	High-grade glioma	21	35	10	30–38						HGG-LGG	0.001	3.29	<0.001	0.60
	Low-grade glioma	9	10	17	2–30						HGG-MENIN	<0.001	4.21	<0.001	0.76
	Meningioma	10	8	7.5	4–15.5						LGG-MENIN	0.609			

IQR = interquartile range, df = degrees of freedom, LGG = low-grade glioma, HGG = high-grade glioma, MTE = mean tissue elasticity, FA = fractional anisotropy, MD = mean diffusivity, AD = axial diffusivity, and RD = radial diffusivity.

**Table 3:** Univariate analysis of Ki-67 groups.

Variable	Ki-67		Wilcoxon–Mann–Whitney Test			
	Low (<10%)	High (>10%)	*U*	*Z*	*p*	*r*
**MTE**	110.34 (28.03)	79.99 (23.15)	48	3.38	<0.001	0.53
**FA**	0.24 (0.16)	0.19 (0.06)	86	2.31	0.020	0.36
**MD**	1.04 (0.47)	1.22 (0.25)	189	0.89	0.373	0.14
**AD**	1.29 (0.36)	1.43 (0.30)	175	0.47	0.649	0.07
**RD**	0.91 (0.53)	1.10 (0.26)	197	1.20	0.238	0.19

Values are expressed as medians and the interquartile range. MTE = mean tissue elasticity, FA = fractional anisotropy, MD = mean diffusivity, AD = axial diffusivity, and RD = radial diffusivity.

## Data Availability

The datasets generated during and/or analysed during the current study are available from the corresponding author on reasonable request.

## Supplementary Materials

The following are available online at https://www.mdpi.com/2076-3425/11/2/271/s1: Figure S1: Boxplots of the mean tissue elasticity values; Figure S2: Diagrams of collinearity diagnosis; Figure S3: Diagnostic plots of regression models; Table S1: Descriptive statistics of tumor regions grouped by histopathologic diagnosis; Table S2: Model summary and coefficients of each regression model; Table S3: Regression model accuracy metrics.
