# Supplementary material for: Is There a Relationship between the Elasticity of Brain Tumors, Changes in Diffusion Tensor Imaging, and Histological Findings? A Pilot Study Using Intraoperative Ultrasound Elastography
Source: Brain Sci. 2021 Feb 21;11(2):271. doi: 10.3390/brainsci11020271 (PMC7924866; doi:10.3390/brainsci11020271)
Supplement: Supplementary file 1 [file brainsci-11-00271-s001.pdf]

Supplementary Materials

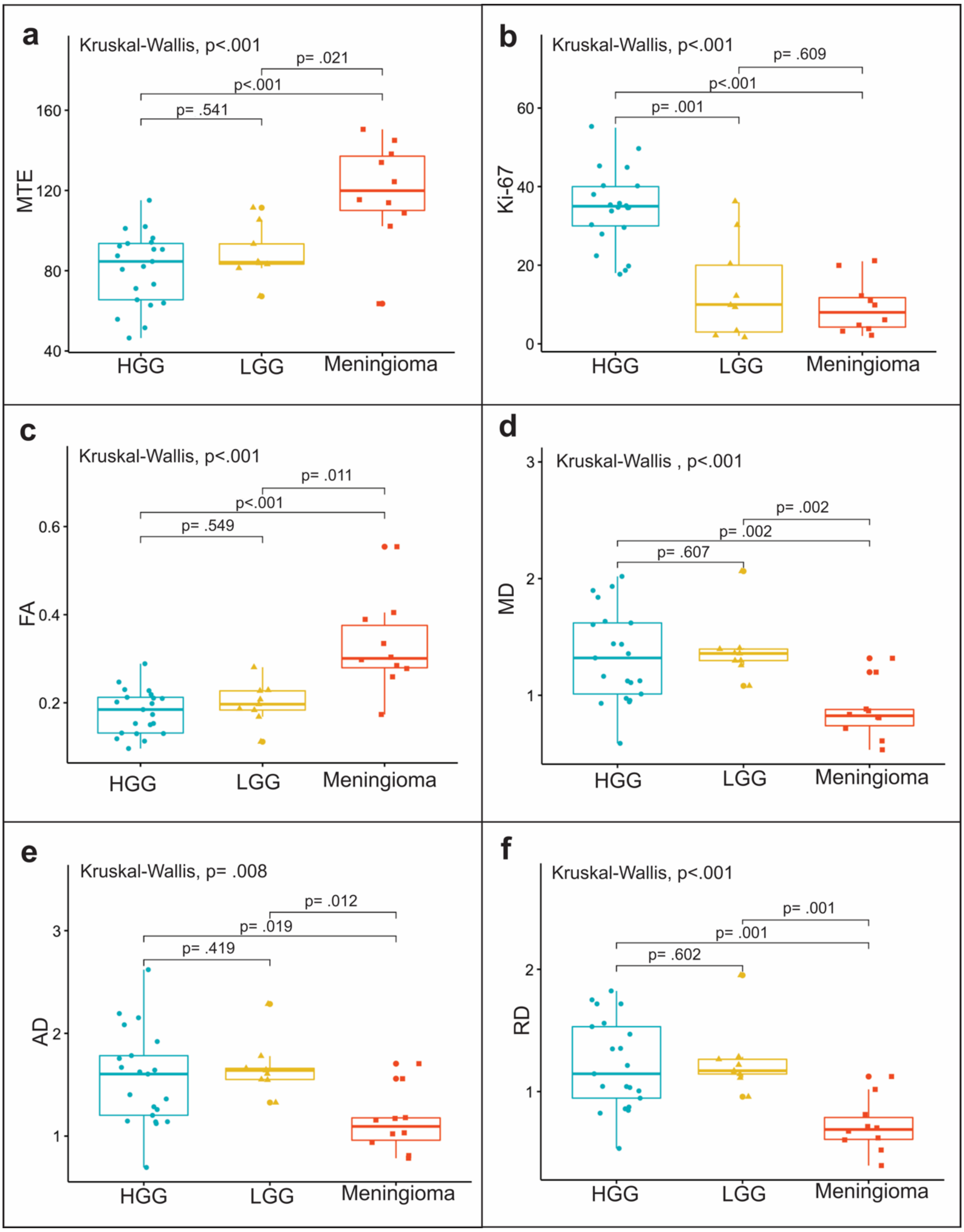

Figure S1. Boxplot showing differences in the median of (**a**) mean tissue elasticity, (**b**) Ki-67, (**c**) fractional anisotropy, (**d**) mean diffusivity, (**e**) axial diffusivity, and (**f**) radial diffusivity, according to histopathology: HGG, high grade glioma; LGG, low grade glioma and meningioma. At the top of each figure, p values for Kruskal-Wallis and post-hoc analysis between group comparisons are presented.

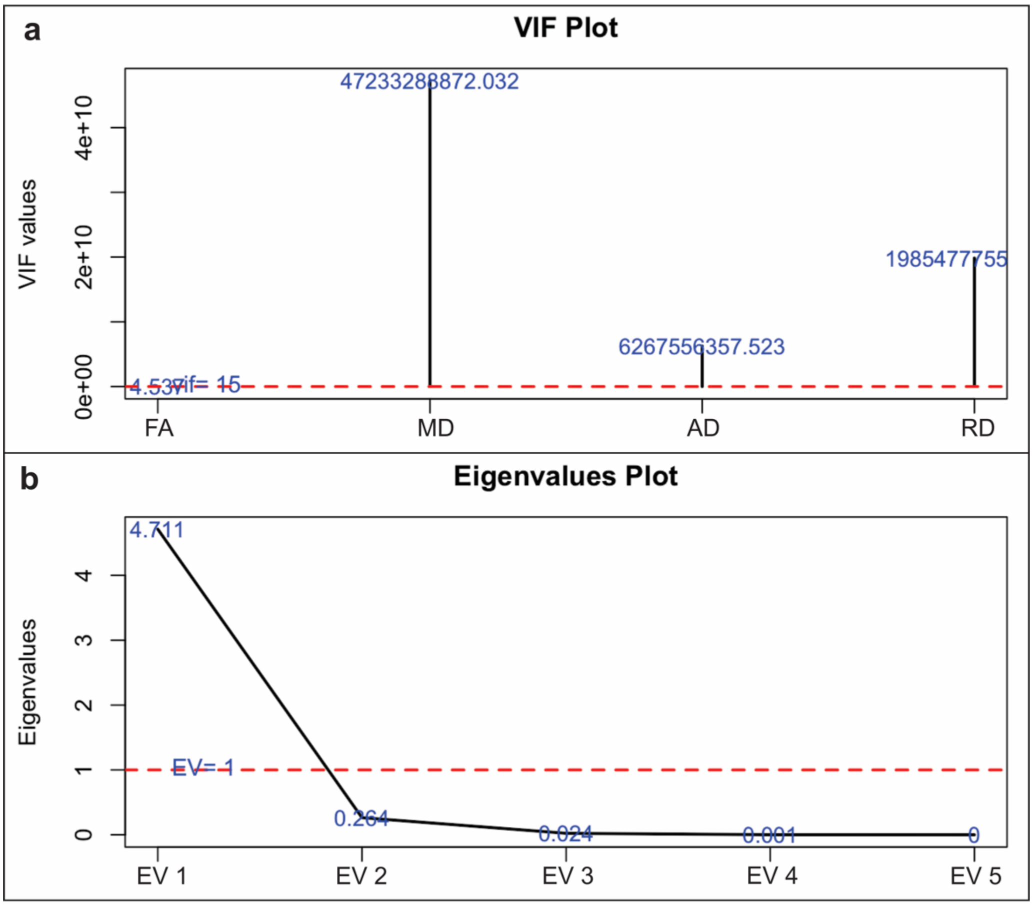

Figure S2. Diagrams of collinearity diagnosis before model building. (**a**) Variance inversion factor (VIF) and (**b**) eigenvalues calculated for diffusion coefficients.

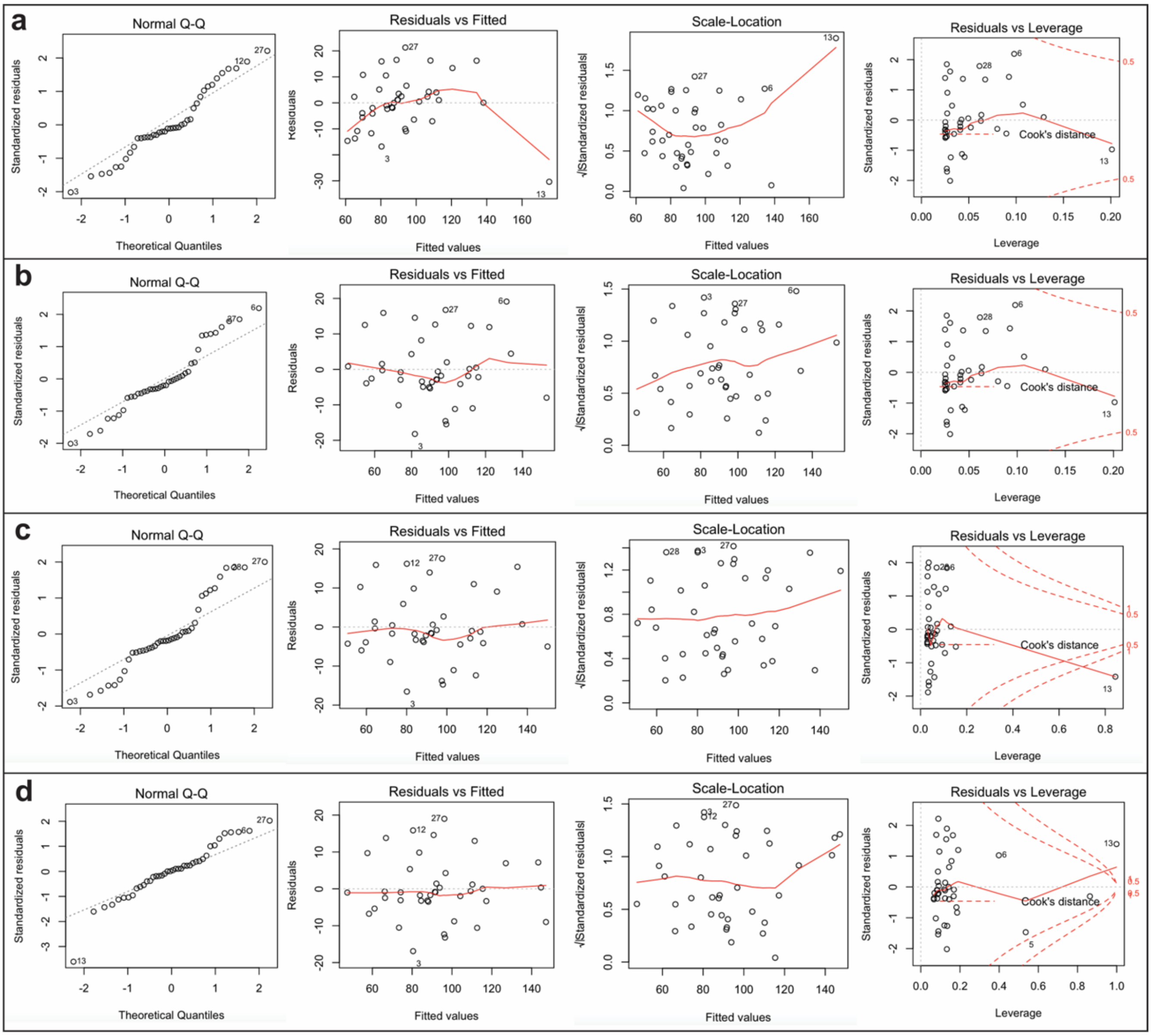

Figure S3. Diagnostic plots of regression models: (**a**) linear, (**b**) logarithmic, (**c**) quadratic polynomial, (**d**) cubic spline. From left to right: normal Q-Q plot, residual vs fitted, spread-location plot and residuals vs leverage.

Table S1. Descriptive statistics of tumor regions grouped by histopathologic diagnosis.

REGION	AP	*n*	MTE	FA	MD	AD	RD
**Core**	High grade glioma	9	61.52 (22.37)	0.14 (0.07)	1.39 (1.11)	1.59 (1.22)	1.32 (1.07)
	Low grade glioma	21	77.66 (21.18)	0.18 (0.02)	1.36 (0.16)	1.67 (0.15)	1.24 (0.19)
	Meningioma	10	132.69 (31.49)	0.32 (0.09)	0.83 (0.11)	1.09 (0.28)	0.70 (0.13)
**Peripheral zone**	High grade glioma	9	101.86 (29.44)	0.21 (0.11)	1.03 (0.23)	1.27 (0.25)	0.93 (0.21)
	Low grade glioma	21	93.40 (18.75)	0.23 (0.06)	1.28 (0.21)	1.56 (0.19)	1.11 (0.09)
	Meningioma	10	113.38 (37.57)	0.29 (0.11)	0.81 (0.22)	1.14 (0.19)	0.64 (0.24)

Values are expressed in medians and interquartile range. MTE = mean tissue elasticity, FA = fractional anisotropy. MD = mean diffusivity, AD = axial diffusivity, RD = radial diffusivity.

Table S2. Model summary and coefficients of each regression model.

	Linear				Logarithmic				Quadratic Polynomial				Cubic Spline			
**Predictors**	**Estimates**	**Std. Error**	**95% CI**	**Statistic**	**Estimates**	**Std. Error**	**95% CI**	**Statistic**	**Estimates**	**Std. Error**	**95% CI**	**Statistic**	**Estimates**	**Std. Error**	**95% CI**	**Statistic**
**(Intercept)**	37.08 ***	4.5	27.97–46.20	8.24	189.20 ***	6.32	176.40–202.00	29.92	6.93	8.18	-9.65–23.51	0.85	47.45 ***	8.35	30.45–64.44	5.68
**FA**	249.54 ***	18.87	211.33–287.75	13.22												
**FA [log]**					61.34 ***	3.89	53.45–69.22	15.75								
**FA[1st degree]**									496.38 ***	61.52	371.72–621.03	8.07				
**FA [2nd degree]**									−429.78 ***	103.52	−639.54 – −220.02	−4.15				
**FA[1st degree]**													17.21	15.36	−14.05–48.46	1.12
**FA[2nd degree]**													27.32 *	10.33	6.31–48.32	2.65
**FA[3rd degree]**													45.49 ***	10.12	24.89–66.08	4.49
**FA [4th degree]**													75.03 ***	14.73	45.07–105.00	5.09
**FA[5th degree]**													132.54 ***	23.74	84.25–180.83	5.58
**FA [6th degree]**													97.21 ***	12.26	72.27–122.15	7.93
**Observations**	40				40				40				40			
**R^2 / R^2 adjusted**	0.821/0.817				0.867/0.864				0.878/0.872				0.890/0.869			
**AIC**	306.7				294.88				293.4				297.5			
**F statistic**	$F(1,38) = 174.82$ ***				$F(1,38) = 248.00$ ***				$F(2,37) = 133.37$ ***				$F(6,33) = 44.28$ ***			

FA = fractional anisotropy. AIC = Akaike information criterion. p values = * $p < 0.05$ ** $p < 0.01$ *** $p < 0.001$.

Table S3. Regression model accuracy metrics.

Estimator	Regression model			
	Linear	Logarithmic	Quadratic Polynomial	Cubic Spline
R^2	0.80	0.79	0.79	0.78
AIC	249.84	238.49	236.94	241.28
MSE	61.96	67.41	71.52	64.51
MAE	6.43	6.42	6.88	6.22
RMSE	7.87	8.21	8.46	8.03
COE	0.57	0.57	0.54	0.58
IOA	0.78	0.78	0.77	0.79
MAPE	0.07	0.07	0.07	0.06

R^2 = *R* squared, AIC = Akaike information criterion, MSE = mean squared error, MAE = mean absolute error, RMSE = root mean squared error, COE = coefficient of efficiency, IOA = index of agreement, MAPE = mean absolute percentage error.
